# Characteristics of Patients Undergoing Revision Thumb CMC Arthroplasty

**DOI:** 10.1016/j.jhsg.2026.101084

**Published:** 2026-08-08

**Authors:** Ethan C. Cossu, Luna Toma, Dafang Zhang, Philip E. Blazar, Leah R. Demetri, Brandon E. Earp

**Affiliations:** ∗ Department of Orthopaedic Surgery, Brigham and Women’s Hospital, Boston, MA; † Harvard Medical School, Boston, MA

**Keywords:** Arthroplasty, Carpometacarpal, Revision, Thumb

## Abstract

**Purpose:**

The purpose of this study was to describe the demographic and operative characteristics of patients undergoing revision thumb carpometacarpal (CMC) arthroplasty and to characterize technique transitions from primary to revision surgery.

**Methods:**

A retrospective review of revision thumb CMC arthroplasty cases was performed within a single health system from August 2015 to August 2025. Cases were identified by querying Current Procedural Terminology code 25447 and screening operative notes for revision-related terminology. For each case, primary and revision operative details were extracted, including reconstruction technique, interposition material, implant(s) used, and trapezium and/or trapezoid excision. Demographics, laterality, dominant hand, smoking status, and diabetes mellitus were recorded.

**Results:**

Forty-two patients who underwent 54 ipsilateral revision thumb CMC arthroplasties were included. The cohort was predominantly women (79%) with a mean age at revision surgery of 62.9 ± 8.6. The median time from primary arthroplasty to first revision was 19 months (interquartile range, 10.9–43.2) with no difference by primary technique. Thirty-five patients (83%) underwent a single revision, six (14%) required two revisions, and one patient underwent seven revisions. The most common primary reconstruction techniques were flexor carpi radialis ligament reconstruction and tendon interposition (38%) and abductor pollicis longus suspensionplasty (33%), while suture-button suspensionplasty was used in only two cases (5%). Suture-button suspensionplasty was the most frequently performed revision technique (33%) followed by abductor pollicis longus suspensionplasty (19%). Approximately 95.2% (40/42) underwent a change in technique at revision. Partial trapezoid excision was performed in 4 of 42 (10%) primary procedures, and trapezoidectomy was performed in 12 of 54 (22%) revision procedures (11 partial, 1 complete).

**Conclusions:**

Ninety-five percent of patients underwent a change in reconstruction technique at revision, and no single primary technique was immune to failure. Trapezoid excision was performed more than twice as often at revision, highlighting the role of adjacent joint pathology in the revision setting.

**Type of study/level of evidence:**

Retrospective review IV.

The thumb carpometacarpal (CMC) joint is a saddle-shaped articulation between the first metacarpal and the trapezium that permits a wide arc of motion, enabling the thumb's essential role in grip, pinch, and opposition.^1^ This mobility, however, comes at the cost of stability, as the joint relies on ligamentous support rather than bony confinement.^2^ As a result, the thumb CMC joint is among the most commonly affected sites of degenerative disease in the upper extremity.^3^ Surgical intervention is considered when nonsurgical management fails.^4^

Multiple surgical techniques have been described, and the optimal procedure remains under debate. Trapeziectomy with ligament reconstruction and tendon interposition (LRTI) with flexor carpi radialis, as originally described by Burton and Pellegrini, has historically been the most widely performed procedure in the United States.^5^^,^^6^ However, a variety of alternative approaches are now in common use, including simple trapeziectomy with or without interposition, abductor pollicis longus (APL) suspensionplasty, suture-button suspensionplasty, suture-only suspensionplasty, arthrodesis, and implant arthroplasty.^6^, ^7^, ^8^ Systematic reviews have not demonstrated clear superiority of any single technique over another with respect to pain relief, functional outcomes, or complication rates.^9^^,^^10^ Consequently, there is substantial variation in technique selection, driven by surgeon training and individual preference.

Revision rates following primary thumb CMC arthroplasty remain relatively low, with reported rates ranging from 0.6% to 5%.^11^, ^12^, ^13^, ^14^ Common failure etiologies include metacarpal subsidence, persistent instability, and scaphotrapezoid arthritis.^15^, ^16^, ^17^, ^18^ However, prior studies have primarily focused on why primary procedures fail rather than on the operative characteristics of the revision itself. The degree to which reconstruction technique, interposition material, and implant selection differ between the primary and revision procedure has not been systematically examined. The purpose of this study is to describe the demographic and operative characteristics of patients who underwent revision thumb CMC arthroplasty within a single health system and to characterize technique transitions from primary to revision surgery.

## Materials and Methods

### Study design and patient identification

An IRB-approved retrospective review of revision thumb CMC arthroplasty cases was performed within our health system from August 2015 to August 2025. Candidate cases were identified by query of an institutional research database using the Current Procedural Terminology code 25447 (arthroplasty, interposition, intercarpal or carpometacarpal joints) and further queried for text containing the terms "revision" or "re-do" within the operative note. This query identified 204 candidate encounters.

All 204 cases underwent operative note review to confirm revision of a prior ipsilateral thumb CMC arthroplasty. One hundred fifty-six patients were excluded because review did not confirm a revision thumb CMC arthroplasty. This yielded 48 confirmed revision thumb CMC arthroplasty cases. An additional 6 patients were excluded, 5 because of lack of an available operative note from the primary procedure, and 1 because of a surgical indication of sequela of traumatic brachial plexus injury rather than thumb CMC osteoarthritis. The final cohort consisted of 42 patients.

### Data extraction

For each patient, demographic variables were recorded, including age at primary surgery, sex, laterality, hand dominance, diabetes mellitus status, and smoking history. The indication for each revision was categorized using the most recent preoperative clinic note, with verification against intraoperative and imaging findings where available. The dates of each primary surgical procedure were documented and the time interval from primary arthroplasty to first revision was calculated in months. Operative details were extracted from each primary and revision operative note, with the primary variable of interest being the reconstruction technique employed at each procedure. Additional operative variables were extracted for each procedure, including interposition material, implant(s) used, use of temporary Kirschner wire (K-wire) fixation, and trapezium and trapezoid status. Trapezium and trapezoid status were classified as complete or partial excision.

### Statistical analysis

Descriptive statistics were used to characterize the study cohort. Continuous variables were reported as mean ± SD or median with interquartile range (IQR). Categorical variables were reported as counts with percentages. The distribution of reconstruction techniques at primary and first revision was compared descriptively.

## Results

### Patient demographics

The final cohort of 42 patients with 54 revision procedures was predominantly women (79%) with a mean age at revision surgery of 62.9 ± 8.6 years. The left side was affected in 27 cases (63%), and 13 patients (30%) underwent surgery on their dominant hand (Table 1).

**Table 1:** Demographic and Clinical Characteristics of Patients Undergoing Revision Thumb Carpometacarpal (CMC) Arthroplasty∗

Variable	Overall (N = 42)
Age at first revision (y), mean ± SD	62.9 ± 8.6
Age at first revision (y), median (IQR)	62 (IQR 58–67)
Time to revision (mo), median (IQR)	19 (IQR 10.9–43.2)
Female, n (%)	33 (79)
Left laterality, n (%)	26 (62)
Dominant hand treated, n (%)	13 (31)
Right-handed, n (%)	34 (81)
Smoking status	
Active, n (%)	2 (5)
Former, n (%)	18 (43)
Never, n (%)	22 (52)
Diabetes mellitus, n (%)	6 (14)

∗ Continuous variables are reported as mean ± SD or median with interquartile range (IQR). Categorical variables are reported as count (percentage).

### Number of revisions and time to first revision

Thirty-five patients (83%) underwent a single revision, six (14%) required two revisions, and one patient (2%) underwent seven revisions. The median time from primary arthroplasty to first revision was 19 months (IQR: 10.9–43.2). The time to first revision was variable across primary technique groups, with no clear pattern by technique.

### Primary reconstruction technique

The most common primary reconstruction techniques were LRTI (38%) and APL suspensionplasty (33%), which together accounted for over 70% of primary procedures (Table 2). Suture-button suspensionplasty was used in only 2 primary cases (5%). The remaining primary procedures consisted of interposition only (10%), internal brace suspensionplasty (7%), flexor carpi radialis suspensionplasty (5%), and suture suspensionplasty (2%). Complete trapeziectomy was performed at the primary procedure in 35 cases (83%), with partial trapezial resection in the remaining 7 (17%).

**Table 2:** Distribution of Reconstruction Techniques Used at Primary and First Revision Thumb Carpometacarpal (CMC) Arthroplasty Among 42 Patients∗

Surgery Technique	Primary n (%)	First Revision n (%)
LRTI	16 (38)	3 (7)
APL suspensionplasty	14 (33)	8 (19)
Suture-button suspensionplasty	2 (5)	14 (33)
Internal brace suspensionplasty	3 (7)	4 (10)
Interposition only	4 (10)	7 (17)
FCR suspensionplasty	2 (5)	3 (7)
Suture suspensionplasty	1 (2)	2 (5)
EPB suspensionplasty	0 (0)	1 (2)
Technique transition at revision		40/42 (95.2%)

APL, abductor pollicis longus; EPB, extensor pollicis brevis; FCR, flexor carpi radialis; LRTI, ligament reconstruction and tendon interposition.

∗ The bottom row indicates the proportion of patients whose reconstruction technique differed between primary and first revision.

### First revision reconstruction technique

At the first revision, the chosen reconstruction technique changed in almost all cases. Suture-button suspensionplasty was the most frequently performed revision technique (33%), followed by APL suspensionplasty (19%), interposition only (17%), and internal brace suspensionplasty (10%). LRTI decreased from 38% at primary to 7% at first revision (three cases). Flexor carpi radialis suspensionplasty (7%), suture suspensionplasty (5%), and EPB suspensionplasty (2%) accounted for the remaining cases.

### Changes in technique from primary to revision

**Figure 1:**
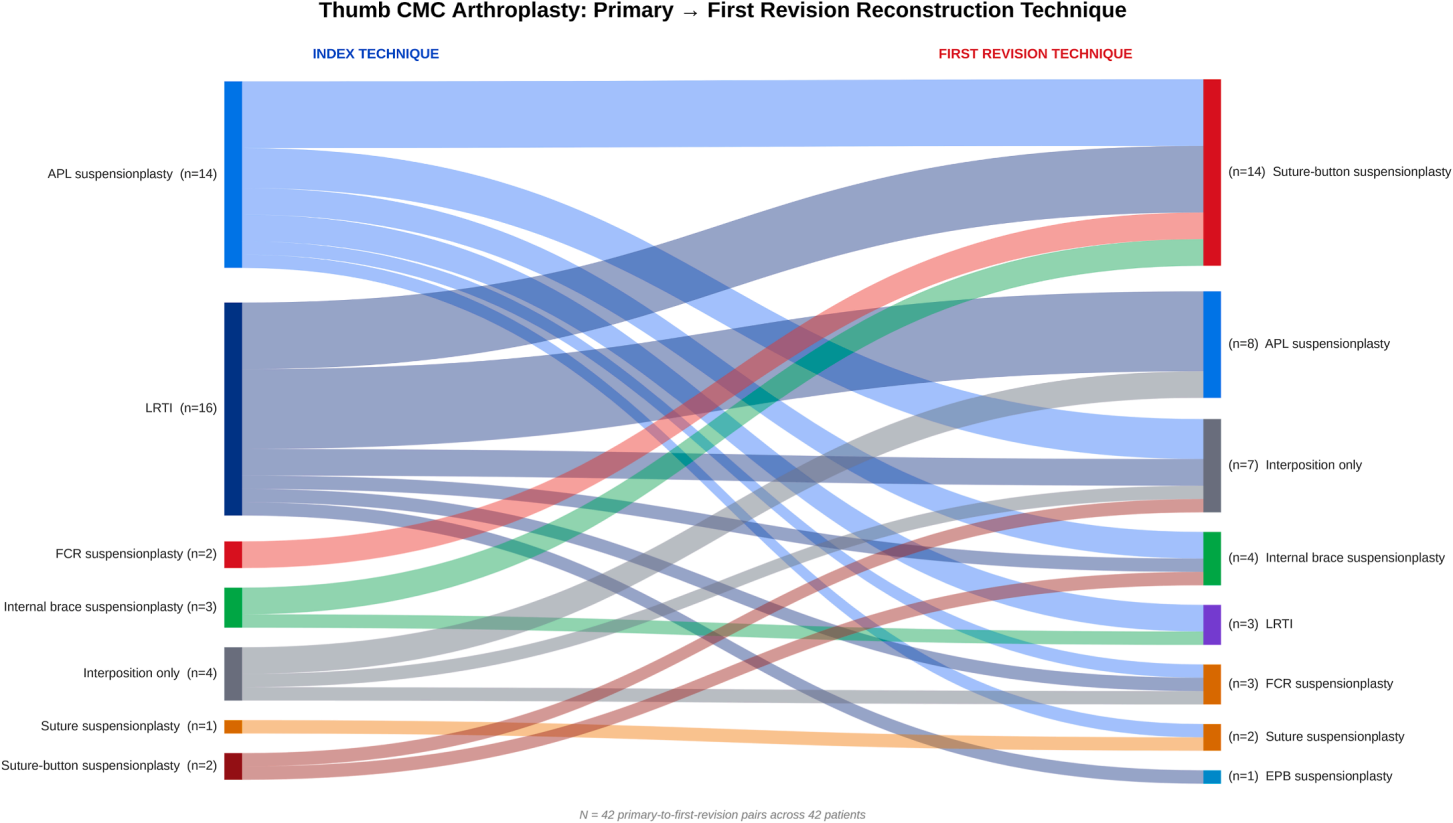
Sankey diagram showing transitions in reconstruction technique from primary to first revision thumb carpometacarpal (CMC) arthroplasty across 42 patients. Primary techniques are listed on the left and first revision techniques on the right, with the width of each band proportional to the number of patients transitioning between categories. Values in parentheses indicate the number of patients in each category. APL, abductor pollicis longus; EPB, extensor pollicis brevis; FCR, flexor carpi radialis; LRTI, ligament reconstruction and tendon interposition.

Forty patients (95.2%) underwent a change in reconstruction technique at revision (Table 2). The two patients who retained the same reconstruction technique had interposition only and suture suspensionplasty at primary. No single dominant pathway from primary to revision technique was identified. Among the 16 patients who underwent LRTI at primary, revision techniques were distributed broadly: 6 to APL suspensionplasty, 5 to suture-button suspensionplasty, and the remaining 5 across four other technique categories. APL suspensionplasty at primary showed a similar pattern of dispersal at revision (Fig. 1).

Several additional operative changes were observed between primary and revision procedures. Interposition material shifted from predominantly tendon autograft at primary (40%) to acellular dermal matrix at revision (31%). Temporary K-wire fixation was used more frequently at revision (31%) than at primary (12%).

### Trapezoid excision

The rate of trapezoid excision was twice as high at revision (22%) compared with primary (10%). Of the 12 revision cases involving trapezoid work, 11 were partial trapezoid excisions and 1 was a complete trapezoidectomy. All four cases at the primary were partial trapezoid excisions.

### Indications

Indications for first revision were categorized into seven groups. Subsidence of the first metacarpal was the most common indication (55%). Persistent pain without an identified mechanical cause was the indication in nine patients (19%). Scaphotrapezoid arthritis was the indication for 7 patients (17%). Less common indications were hardware failure or intolerance, incomplete trapezial excision, metacarpophalangeal joint hyperextension or instability, and persistent thumb instability (9%).

## Discussion

In this single-system retrospective review of 42 patients undergoing revision thumb CMC arthroplasty, 95.2% of patients underwent a change in reconstruction technique between their primary and first revision procedure. The demographic profile of this cohort was consistent with that of the general thumb CMC osteoarthritis population, with a predominance of women and a mean age in the sixth decade.^7^ At the primary procedure, LRTI and APL suspensionplasty were the most commonly performed techniques compared to suture-button suspensionplasty at revision. Partial trapezoid excision was performed more than twice as frequently at revision compared with the primary procedure. Together, these findings demonstrate the need for familiarity with multiple techniques to address the variability in pathoanatomy after failed primary surgery.

Prior series have emphasized that the choice of revision procedure depends on both the underlying cause of failure and the technique used at the primary operation.^17^ Revision surgery is frequently performed in the setting of metacarpal subsidence, persistent instability, or scaphotrapezoid arthritis, each of which may require a distinct operative approach.^17^^,^^19^ Subsidence may need to be addressed to stabilize the thumb metacarpal base and anatomic changes also impact surgical technique, including prior tendon harvest and implant positioning.^20^ The availability of newer devices over the study period provides additional options for reconstruction. Suture-button suspensionplasty was described as a novel technique in 2010, and its emergence during the later years of our study may partially account for its high frequency at revision compared with primary.^6^^,^^21^

Another notable finding of this study was the more than 2-fold increase in trapezoid excision at revision compared with the primary procedure. This pattern suggests that scaphotrapezoid pathology may be underrecognized at the time of the primary operation or may develop over time and become symptomatic only after the initial procedure. Prior work indicates that scaphotrapezoid arthritis is a common coexisting finding in patients with thumb CMC osteoarthritis, with intraoperative identification reported in 31% to 62% of cases.^20^^,^^22^ This finding is further supported by a recent revision series, in which unrecognized scaphotrapezoid arthritis was among the most common pathologies identified at reoperation.^19^ Relevant scaphotrapezioltrapezoid arthritis can be difficult to confirm on radiographs preoperatively, and intraoperative visual assessment of degenerative changes of this joint is critical. This study has several limitations. As a single-system retrospective review, the observed patterns may reflect institutional practice and surgeon preference at this health system rather than broader regional or national trends, and some patients may have had subsequent procedures elsewhere that may not be captured in our cohort. Functional outcomes and patient-reported measures were not collected, and therefore, the clinical outcomes were not assessed. Despite these limitations, this study provides a structured characterization of the operative relationship between primary and revision thumb CMC arthroplasty within a single health system and offers a framework for future investigation into technique selection at revision.

These findings suggest that revision thumb CMC arthroplasty may be a fundamentally different operative problem than the primary procedure. The findings of this study reflect practice patterns at a single health system. They describe how surgeons at this institution approached revision thumb CMC arthroplasty and should not be interpreted as generalizable recommendations for surgical management. Surgeons will benefit from a broad knowledge of techniques and implant options in order to tailor their reconstruction technique in a revision setting. This study did not assess clinical outcomes and cannot establish which techniques perform best. Future work could identify specific failure modes with revision technique selection and evaluate functional outcomes following revision thumb CMC arthroplasty. Multi-institutional studies may further clarify whether the technique patterns observed here reflect a broader operative paradigm or institutional practice.

## Conflicts of Interest

No benefits in any form have been received or will be received related directly to this article.
